# Orienteering in Knowledge Spaces: The Hyperbolic Geometry of Wikipedia Mathematics

**DOI:** 10.1371/journal.pone.0067508

**Published:** 2013-07-03

**Authors:** Gregory Leibon, Daniel N. Rockmore

**Affiliations:** 1 Department of Mathematics, Dartmouth College, Hanover, New Hampshire, United States of America; 2 Department of Computer Science, Dartmouth College, Hanover, New Hampshire, United States of America; 3 THe Neukom Institute for Computational Science, Dartmouth College, Hanover, New Hampshire, United States of America; 4 The Santa Fe Institute, Santa Fe, New Mexico, United States of America; University of Vermont, United States of America

## Abstract

In this paper we show how the coupling of the notion of a *network with directions* with the adaptation of the *four-point probe* from materials testing gives rise to a natural geometry on such networks. This *four-point probe geometry* shares many of the properties of hyperbolic geometry wherein the network directions take the place of the sphere at infinity, enabling a navigation of the network in terms of pairs of directions: the geodesic through a pair of points is oriented from one direction to another direction, the pair of which are uniquely determined. We illustrate this in the interesting example of the pages of Wikipedia devoted to Mathematics, or “The MathWiki.” The applicability of these ideas extends beyond Wikipedia to provide a natural framework for visual search and to prescribe a natural mode of navigation for any kind of “knowledge space” in which higher order concepts aggregate various instances of information. Other examples would include genre or author organization of cultural objects such as books, movies, documents or even merchandise in an online store.

## Introduction

Network navigation generally lacks anything like a compass. Movement occurs more or less in a point-to-point fashion, perhaps with the intent of passing through a particular landmark or in such a way as to optimize some sort of objective function such as path length. Unless embedded in some ambient space (e.g, a Euclidean space via multidimensional scaling [1]) which itself contains global reference points, a notion of global direction is not to be found.

In contrast, in this paper we take the point of view that in many cases, a system or network under consideration often has natural directions. This makes it possible and useful to enable a means of “orienteering” on a network. The mathematics webpages of Wikipedia (“MathWiki”) provide a good example [2]. These pages effectively realize the “space of mathematics” and their navigation is an act of exploration of a world of mathematical ideas. Abstractly, natural directions in this space are broad overarching concepts such as “geometry” or “probability.” A curious explorer of the world of mathematics “coming from calculus” might wish to navigate from a particular concept, say The Fundamental Theorem of Calculus, in the “direction of geometry” to discover related geometric concepts and applications. Merchandise spaces are another source of good examples. For instance, an explorer of the Netflix movie database (realized in some fashion as a network) looking to broaden his or her movie viewing might want to navigate in a particular “direction” of some other genre or subject from a different genre and initial reference point.

In this paper we show how directions on a network can be encoded as a preferenced set of vertices. Such a *network with directions* is much like the unit disk with boundary that forms the point set for a model for hyperbolic geometry, often known as *the Poincaré disk* or its conformal equivalent, the *upper half plane* (in the complex plane), obtained by sending a point on the disk's boundary to infinity [3]. In these examples the boundary acts as a set of “points at infinity.” Our set of directions play an analogous role and similarly, give rise to a natural analogue of the hyperbolic metric. The construction is based on an idea from materials testing in which a *four-point probe* (FPP) is used to measure the resistivity of a material sample (see e.g., [4]). The FPP functions by sourcing current to two “outer probes” and then the voltage across the two “inner probes” is measured. This idea makes sense in any situation in which there is a notion of electric potential. In particular, this is a well known framework for the analysis of random walks on networks [5] and is the context in which we describe the FPP in this paper (although others are possible as well). Random walk-based analyses also underlie various other structural measures for networks such as spectral clustering [6], the PageRank measure of vertex importance [7], [8], and betweeneness [9]. A potential theoretic framework has also been used effectively in the study of networks through the use of *commute-time* as a means to achieve a different sort of metric embedding [10]–[12].

Our discovery of the hyperbolic nature of this *four-point probe geometry* of a network with directions as an actual metric structure on a network is very different, both in spirit and mechanics, from the way in which the adjective *hyperbolic* has been used previously to describe networks [13], [14]. In particular, the metric we construct, in combination with a declared set of directions, gives rise to a notion of geodesic, or more precisely, *geodesic bundle*, that describes an optimal path from one vertex to another, with the requirement that the navigator specify both the direction from which he or she is moving as well the direction in which he or she would like to move. This generates a “best” path from where you have been to where you want to go.

While for problems of simple resource delivery, the standard path-length metric may be the most appropriate, there may be others in which this more “discursive” (in terms of path length) geodesic is a better fit. We suggest that *knowledge networks*, such as Wikipedia, may be such instances. In this situation, the existence of highly connected hub-like portals give the network a very small path-length diameter that can be highly discontinuous in terms of conceptual content. Instead, our four-point probe geometry finds geodesics that appear to link a series of closely related ideas, whose navigation provide a much more natural sense of conceptual connection than that produced by the goal of efficient path length navigation. Other approaches to the efficient navigation of Wikipedia (and thus, “Wikipedia-like” networks) have also recently been suggested [15], [16], while search in other and related (e.g., power-law networks, small world networks, social networks) has been taken up in the papers [17]–[21] environments. The kind of oriented organization proposed here also enables new visualizations for knowledge networks, creating interesting and thematically consistent notions of neighborhoods of ideas.

We anticipate that this geometric network model may prove useful for new notions of network search and exploration and in particular, a model for visual search of a network such as the WWW or online warehouses.

## Results

Our main results are two-fold. We adapt the notion of the four-point probe to a network with directions to show that this gives rise to a pseudometric on the vertices (recall that a pseudometric differs from a metric only in that the distance between two non-identical points can be zero). We further show that this pseudometric behaves much like the hyperbolic metric. The pseudometric enables us to define the notion of geodesic bundle on a network, which behaves much like a geodesic in the familiar models of hyperbolic space. Proofs are left for the Methods section. We then apply these ideas to the MathWiki to see how the hyperbolic structure manifests itself therein and give example geodesics (Tables 2 and 3) to show the way in which this geometric framework presents a new framework for network navigation that may be better attuned to idea exploration.

### Networks with directions

We define a *network with directions* (NWD) to be a network with a privileged subset of vertices called *directions* which we will denote as 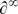. We denote the complement of 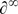 as 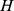. In some instances the directions of the NWD can be thought of as a natural boundary of the system (the notation purposely recalls that of the upper half-plane model of hyperbolic geometry). In others, the directions have a natural interpretation as a set of directions for orientation which can provide an interesting and useful heuristic for navigating the network. Note that in some cases (e.g., the MathWiki) the directions already come as vertices in the space. In other situations we may be required to create a new vertex to encode a direction. We also assume that the NWD has a weight (adjacency) matrix 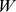 from which we can form a Markov chain with transition matrix 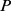 formed by normalizing the row sums of the weight matrix to be one. We further assume that for a NWD this chain is ergodic. See [22] for Markov chain basics.

A NWD has a natural pseudometric that can be derived from the *four-point probe* which is an engineering tool used to find imperfections and cracks in materials [4]. Roughly, the four-point probe works by using a battery to create charges of 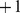 at a point 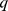 and of 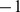 at a point 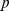, and then using a probe to measure the potential difference between two other points 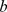 and 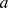 which we denote as 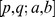. As we show in the Methods section, the four-point probe is easily conceptualized and realized on a NWD (or in any setting where the definition of a potential makes sense), and can be used to explore the NWD's geometry. The use of ideas from electrical networks to analyze topological networks is well known [5].

The distance 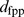 defined by the four-point probe is such that given a pair of points 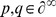, then for any points 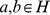 we have
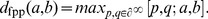
(1)


Theorem 1 in the Methods section assures us that 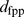 forms a pseudometric. The distance 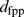 behaves mathematically in many ways just as we would expect a discretization of the hyperbolic metric to behave. Theorem S2 in Text S1 demonstrates that given a disk in the Riemann sphere, the construction of 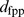 with the four-point probe gives the hyperbolic metric in the disk and it forms the familiar model of the hyperbolic plane known as the Poincaré disk [23]. We call the NWD with 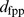 the *FPP geometry on the NWD.*

**Figure 1:**
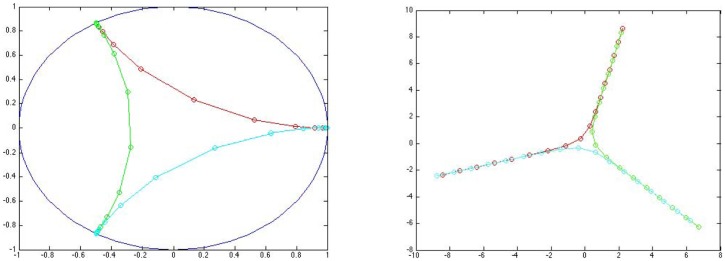
Hyperbolic triangle embedding comparisons. Comparison of ideal triangle in the conformal Poincaré model (left) with MDS embedding of the (truncated) triangle with indicated points on the triangles's boundary embedded respecting distance (right). The relevance is that to embed our networks we use the 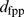 metric and the MDS into a Euclidean space, and as such our representations will be attempting to mimic the distances (as best as possible) and not the angle as is the case in the more familiar Poincaré and upper half space representations of hyperbolic geometry.

**Figure 2:**
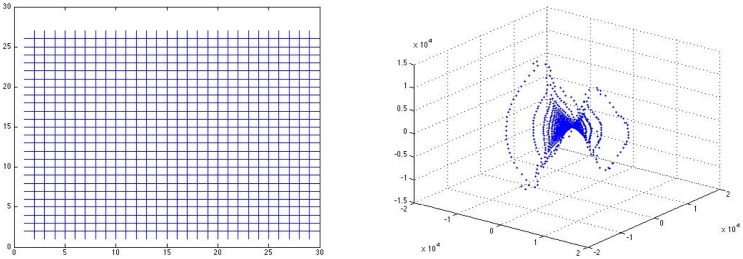
Square grid as a network with directions. The lefthand figure shows a square grid with obvious boundary given by the collection of vertices with less than four neighbors. On the right is the three-dimensional MDS embedding of the hyperbolic metric 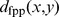 on the chain with 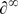 given by the boundary. Notice the saddle point structure of the embedding, consistent with a hyperbolic geometry.

For visualization of a NWD we will use multidimensional scaling (MDS) [1] applied to 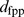 matrix of vertices in the network to embed the vertices in a Euclidean space. The MDS construction aims to preserve distances as best as possible, while the Poincaré model preserves angles but badly distorts distances. To gain some intuition consider Figure 1, wherein we see the difference in appearance of a truncated (i.e., necessarily not extended out to infinity) ideal triangle in the Poincaré model (on the left) that we use for our schematics, and what that triangle looks like if we embed the indicated points using the hyperbolic distances via MDS (on the right). For comparison, in a second example, we consider a simple NWD given by the square grid network with boundary (directions) shown on the left in Figure 2. Therein, we assign unit weights to the edges and let 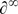 be the set of vertices connected to fewer than four other vertices (the obvious boundary of vertices of the grid) so that 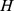 comprises the remaining (interior) points. On the right in Figure 2, we show the three-dimensional MDS embedding of 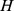 using 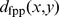. Note the nice negative curvature saddle surface that we would anticipate for a hyperbolic geometry [23].

**Figure 3:**
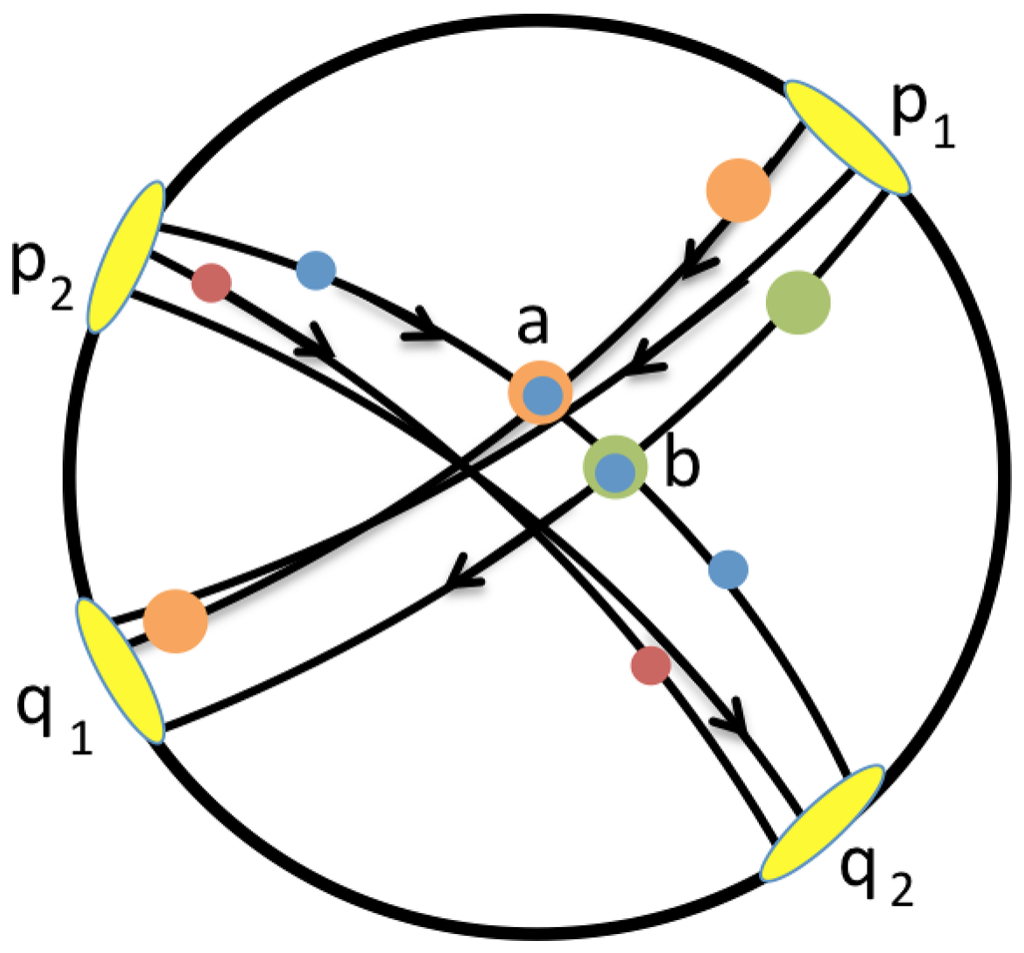
Schematic of a pair of geodesic bundles. We have that 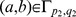, but, although there are points in the form 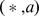 and 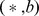 in 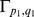, we see that 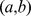 is not in 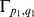.

For our needs, the most important construction in hyperbolic geometry is that every ordered pair of points 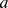 and 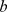 determines a unique oriented geodesic from 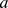 to 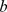 which can then be extended to hit a unique pair of points in 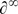. More precisely, calling this unique pair of points at infinity 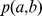 and 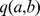, they are determined by
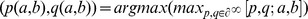



(see Theorem S1 in Text S1). Conversely given a pair of directions 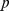 and 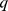 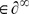 then we can consider the sets of pairs of points in 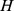 that give rise to them:
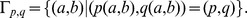
While in the case of hyperbolic space 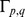 would be a unique geodesic, our NWD behaves like a discretization of hyperbolic space, so a vertex at infinity is effectively like a subset of points at infinity in hyperbolic space. Thus in the NWD case we obtain a *geodesic bundle*, 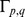, of oriented geodesics that connect from points in 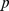 to points in 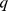. That is, 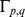 is not necessarily itself a single geodesic. In Text S1 we show how the definitions above are entirely consistent with the analogous constructions for hyperbolic space. Our use of the term bundle is more closely aligned with its use in relativistic cosmology (see e.g., [24]) versus the more common mathematical use of the term (e.g., tangent bundle) from geometry. See Figure 3 for a schematic illustrating the notion of oriented geodesic bundle.

Some facts that would apply to geodesics in hyperbolic geometry still hold for our geodesic bundles 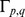 in a NWD. For example we have the consistency relation that if 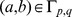 and 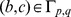 then 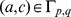 which one would expect for oriented geodesic bundles (see the Methods section, Theorem 2). On the other hand, since we are dealing with geodesic bundles and not geodesics, if 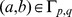 and 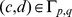 then there is no reason for 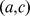 or 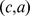 to be in 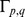.

### The hyperbolic geometry of MathWiki

We now illustrate the FPP geometry for a NWD in the example of the MathWiki [2] the subset of the WWW and Wikipedia determined by the subset of webpages of Wikipedia that are devoted to mathematics (see Data Sources for details). In the MathWiki we use the “List of” pages to capture the notion of direction. This could also be done for the various other portals of Wikipedia. Table 1 shows the “List of” pages in the MathWiki. More generally, any categorical grouping of the set of entities in a space could provide a natural set of orientations for a network space. Note that such a grouping need be neither complete nor non-overlapping.

**Table 1:** The points at infinity in the MathWiki Space.

The ‘List of’ pages at ∞	
List of Abstract Algebra Topics	List of Curve Topics
List of Triangle Topics	List of Mathematical Topics in quantum theory
List of Lie Group Topics	List of algebraic_coding_theory Topics
List of Complex Analysis Topics	List of Set Theory Topics
List of Basic Probability Topics	List of Fourier Analysis Topics
List of general Topology Topics	List of Algorithm General Topics
List of geometry Topics	List of Partial Differential Equation Topics
List of numerical Computational Geometry Topics	List of Topology Topics
List of Geometric Topology Topics	List of Group Theory Topics
List of Computer Graphics and Descriptive Geometry Topics	List of Multivariable Calculus Topics
List of Partition Topics	List of Differential Geometry Topics
List of Statistical Topics	List of Variational Topics
List of Stochastic Processes Topics	List of Permutation Topics
List of Linear Algebra Topics	List of Algebraic Topology Topics
List of Calculus Topics	List of Homological Algebra Topics
List of Exponential Topics	List of Number Theory topics
List of Commutative Algebra Topics	List of Recreational Number Theory Topics
List of Computability and Complexity Topics	List of Basic Algebra Topics
List of Boolean Algebra Topics	List of Mathematical Logic Topics
List of Representation Theory Topics	List of Integration and Measure Theory Topics
List of Factorial and Binomial Topics	List of String Theory Topics
List of Numerical Analysis Topics	List of Topics Related to pi
List of Real Analysis Topics	List of Mathematical Topics in Relativity Topics
List of Knot Theory Topics	List of Trigonometry Topics
List of Convexity Topics	List of Algebraic Number Theory Topics
List of Functional Analysis Topics	List of Numeral System topics
List of Probability Topics	List of Combinatorial Computational Geometry
List of Dynamical Systems and Differential Equations Topics	List of Polynomial Topics
List of Graph Theory Topics	List of Order Theory Topics
List of Mathematical Topics in Classical Mechanics'	List of Circle Topics
List of Harmonic Analysis Topics	List of Algebraic Geometry Topics

These are the “List of” pages that make up the set of directions in the MathWiki viewed as a network with directions.

Various choices could be made in forming the actual transition matrix of the chain. During our analysis, we explored both treating the links as directed and undirected, and the resulting geometries were qualitatively similar though somewhat different in the details. We choose to present the result from the undirected analysis, as the results appeared a bit more natural. (Arguably, this makes sense: to get to Quantum Field Theory from the calculus it is necessary to transition through Hilbert Space, even if the links all flow the other direction.) We enforced that all transitions were proportional to the available links subject to a 1/50 chance of transitioning to a “List of” page at 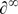 (when that was possible), and that at every page there is a 1/200 chance of starting over and going to the “List of all Mathematics Articles”. This last condition insures ergodicity of the chain. Admittedly these weights are arbitrary (indeed, not unlike a first guess at 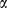 in PageRank [7]), but they are conceptually reasonable. The best way to make these choices would be to utilize an appropriate objective function (as discussed in the Discussion section).

**Figure 4:**
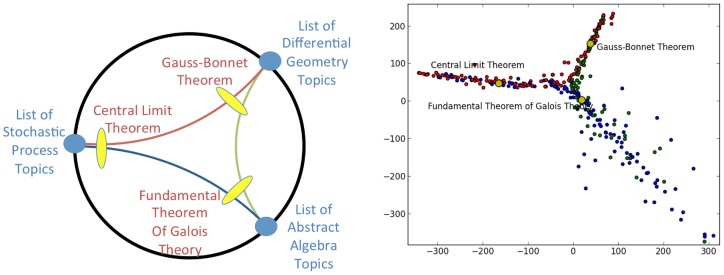
MathWiki Space triangle Example 1. In this example we see a triangle in the MathWiki space, determined by the vertices that correspond to the Math Wiki pages for The Central Limit Theorem, The Fundamental Theorem of Galois Theory, and The Gauss-Bonnet Theorem. We have extended the geodesic bundles between the vertices towards infinity. On the left is a schematic showing us the points at infinity involved (as the “List of” pages) and how this triangle might appear in the Poincaré disk model. When we view the actual network on the right we use the 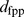 metric and MDS to place these vertices into a two-dimensional Euclidean space. Hence, the representation in that figure will look similar to the MDS of a triangle in hyperbolic space and not the conformal representation in the Poincaré Disk model.

Figure 4 shows an example of a triangle in this space, constructed on the vertices (Wikipedia pages) corresponding to The Central Limit Theorem [25], The Fundamental Theorem of Galois Theory [26], and The Gauss-Bonnet Theorem [27]. These are respectively, famous theorems from probability, algebra, and geometry. While it is possible that the three pairs of points could determine three non-overlapping pairs of directions, in fact, in this case, the three pairs of directions determined by the three pairs of points are made up of only three directions – i.e., the points lie a single ideal triangle whose vertices are the “List of Stochastic Process Topics”, “List of Abstract Algebra Topics”, and “List of Differential Geometry Topics” pages. This is indicated by the lefthand side of Figure 4. We construct a sampling of the edges utilizing the relevant geodesic bundles as follows: for each of the three pairs of vertices 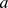 and 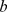 among the concepts of interest we selected a set of points 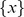 on the geodesic bundle by randomly selecting pairs in 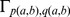 of the form 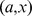, 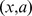, 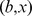, or 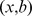. Table 2 lists some of the points involved in the segment from 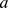 equal to The Fundamental Theorem of Galois Theory to 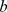 equal to The Gauss-Bonnet Theorem. To isolate this segment we further require that our pairs are in the form 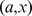 or 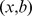 subject to the condition that 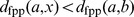 or 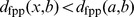 respectively (i.e., we require that 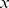 is “between” 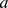 and 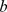). To move along the geodesic from 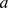 to 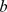 using these points, we compute the distance from 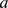 to 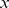 as 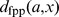 and 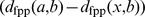 for the cases 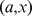 and 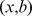 respectively. This allows us to create a list of points ordered by their distance (a traversal) from 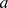 to 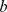, of which Table 2 is a particular example.

**Table 2:** Fundamental Theorem of Galois Theory to Gauss-Bonnet Theorem.

FPP-Geodesic	Path Length Geodesic
Fundamental Theorem of Galois Theory	Fundamental Theorem of Galois Theory
Pfister Form	Fundamental Theorem of Algebra
Prufer Rank	The Gauss-Bonnet Theorem
Tensor Product of Quadratic Forms	
Fundamental Domain	
Modular Symbol	
Unfolding (functions)	
Kuga Fiber Variety	
Minkowski-Hlawka Theorem	
Fuchsian Model	
(GX)-manifold	
Poincare Model	
Riemann Manifold	
Gromov's Compactness Theorem (topology)	
Cayley Surface	
Prescribed Scalar Curvature Problem	
Connector (mathematics)	
Calculus on Manifolds	
The Gauss-Bonnet Theorem	

In the column on the left we see the FPP-geodesic that navigates in the MathWiki from ‘The Fundamental Theorem of Galois Theory’ to the ‘The Gauss-Bonnet Theorem’ (with respective associated directions – i.e., “List of” pages – ‘List of Algebra Topics’ and ‘List of Geometry Topics’) and on the right we see the path length geodesic between these two pages. Note that the FPP-geodesic results in a more conceptually gradual path than the path length optimized route.

**Table 3:** Geodesic from Gauss-Bonnet Theorem to the Central Limit Theorem.

FPP-Geodesic	Path Length Geodesic
The Gauss-Bonnet Theorem	The Gauss-Bonnet Theorem
Calculus on Manifolds	Measure (mathematics)
Nonmetricity Tensor	Gibbs Measure'
Autoparallel	Central Limit Theorem
Riemann Manifold	
Last Geometric Statement ofJacobi	
Peetre Theorem	
Uniformization	
Distortion (mathematics)	
Statistical Manifold	
Minkowski Distance	
Invariant Measure	
Index Set	
Self-dissimilarity	
Realization (probability)	
Stationary distribution	
Information Projection	
Martingale Difference Sequence	
Slepian's Lemmae	
Minimal Entropy Martingale Measure	
Limit Theorem	
Central Limit Theorem	

In the column on the left we see the FPP-geodesic that navigates in the MathWiki from the ‘The Gauss-Bonnet Theorem to the ‘The Central Limit Theorem’ (with respective associated directions – i.e., “List of” pages – ‘List of Geometry Topics’ and ‘List of Stochastic Processes’) and on the right we see the path length geodesic between these two pages. Note that the FPP-geodesic results in a more conceptually gradual path than the path length optimized route.

In the righthand side of Figure 4 we show an MDS embedding of points in the three sets of geodesic bundles, further augmented by extending each set toward the appropriate point at infinity. The points are color-coded according to the geodesic bundle of which they are a part. Notice that we find a set of points that resembles a sampling from an ideal triangle in hyperbolic space (as discussed in Figure 1).

**Figure 5:**
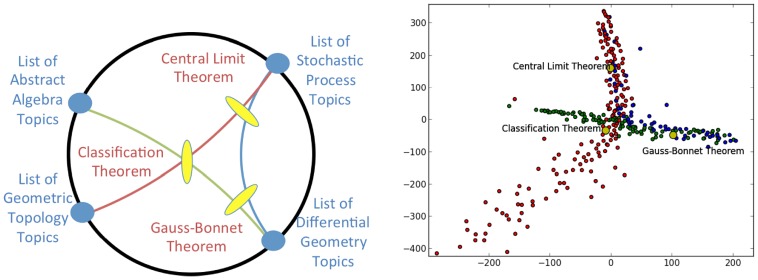
MathWiki Space triangle Example 2. Here we have modified the example of Figure 4 by replacing the node (MathWiki page) for The Central Limit Theorem with that of Classification Theorem and leaving the other two nodes the same (given by the pages for The Fundamental Theorem of Galois Theory and The Gauss-Bonnet Theorem). Again we extend the geodesic bundles between the vertices towards infinity. On the left is a schematic showing us the points at infinity involved (as the “List of” pages) and how this triangle might appear in the Poincaré disk model. When we view the actual network on the right we use the 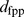 metric and MDS to place these vertices into a two-dimensional Euclidean space. Hence, the representation in that figure will look similar to the MDS of a triangle in hyperbolic space and not the conformal representation in the Poincaré Disk model. Note that in this example, there are four directions (points at infinity) involved, reflecting the difference in (conceptual) proximity to The Fundamental Theorem of Galois Theory and The Gauss Bonnet Theorem of The Classification Theorem versus that of the Central Limit Theorem.

Figure 5 shows a different scenario. In this case we replaced the use of the The Fundamental Theorem of Galois Theory with Classification Theorem [28], a page dedicated to explaining classification theorems. This concept is very central to mathematics and should be close to both The Gauss-Bonnet Theorem and The Central Limit Theroem as they play intimate roles in the classification of constant curvature surfaces and probability distributions, respectively. Unlike the previous case, here the three pairs of directions determined by the three pairs of points give a total of four distinct directions. This is indicated on the lefthand subfigure. In this case, the three points do not determine a unique ideal triangle. The righthand subfigure is again a sampling of the three geodesic bundles, extended toward infinity, color-coded accordingly. As suggested by the righthand figures in Figures 4 and 5, the difference effectively derives from that of the former triangle being determined by three “very distant” concepts (points) while the latter is determined by choosing a point (The Classification Theorem) that is closer to the other two points, notions that are consistent with the ways in which we conceive of these triples from a mathematical point of view.

## Discussion

A NWD and accompanying pseudometric can be constructed for any spaces in which the notion of a potential makes sense. In both of our examples, a basic Markov chain with natural structural boundary and the MathWiki, the set of directions come as part of the network of interest. In the MathWiki the “List of” pages are a part of the network, but they are also just an aggregation of webpages. Note that the “List of” pages neither comprise a complete nor disjoint grouping of the MathWiki. This idea, extended to other subsets of the web or networks generally would produce other kinds of geometries and other kinds of geodesics. Such modifications would depend on the goal of the web exploration. As Figures 4 and 5 indicate, the FPP geometry suggests a natural framework for web visualization: a query need not return a simple list of web pages of interest, but rather, the elements of that list embedded in the FPP geometry relevant to the search which could be navigated according to some kind of cartographic user interface such as the Google Maps API [29]. The networks and the geodesics also, of course, depend quite a bit on the edge weights. It is possible that it would make sense to take into account things like user metadata or actual usage patterns to continuously update the metric as a given network is used.

It is also possible to add a set of directions to the chain or network of interest. For example, in the case of applying these ideas to a corpus of documents, points at infinity could be added to reflect genre or authorship, or topic or style (either in an annotated or quantitatively derived [30], [31] description). Similar extensions might be executed for the analysis of other kinds of cultural artifacts, such as movies, or for the navigation of a product space such as encompassed by merchandising gateways like Amazon, with the attendant possibilities mentioned above for visual search of such spaces. In these cases, the hyperbolic geometry could be used to “nudge” a user from one category to another: given a user starting at a particular product 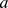, then given a paradigmatic product 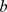 in a class of interest, the associated pair of “from” and “to” directions 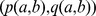 are determined, from which points close to 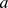 moving toward 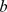 could be determined.

Choice of graph weights is also an important consideration. The clearest method to make such choices would be to decide on an objective function and learn the optimal parameters with regard to this objective. A nice example of this is given in [15] where parameters are learned to find several different geometries based on minimizing the path length between a pair of vertices in Wikipedia by utilizing decentralized search algorithms (see e.g., [17]) as it is related to the Wikispeedia game [32].

Shortest path geometries are terrific when the goal is to efficiently get somewhere or propagate a piece of information. Our belief is that the geometry presented here will likely be better suited to situations in which there is something to be gained by actually having a more discursive (in terms of path length) traversal between two points. For example, consider a MathWiki scenario in which someone knows the Calculus and would like to get a feel for Topological Quantum Field Theory. In such a case, a direct path in the MathWiki space might not be the best way to incrementally prepare someone with a calculus background for the rigors of Topological Quantum Field Theory.

Explicit examples of such comparisons are given in Tables 2 and 3. Clearly, the two-step path from ‘The Fundamental Theorem of Galois Theory’ to ‘The Gauss-Bonnet Theroem’ via the ‘The Fundamental Theorem of Algebra’ (shown in the righthand column of Table 2) is very efficient but does not capture in any sense the journey between these pieces of knowledge. This is also clearly true for the path length geodesic path from ‘The Gauss-Bonnet Theorem’ to the ‘The Central Limit Theorem’ via ‘Measure’ and ‘Gibbs Measure’ shown in the righthand column of Table 3. Moreover, the examples given here are indicative of the structure of the MathWiki space – that is, the (hyperlink) path distance between any two pages in the space is generally quite small due to the existence of the aggregating “List of” pages. Even when ignoring the “List of” pages we find that greater than 98.4% of the space is in the component that contains the page ‘Real number’ [33] and 99.8% of the pages in this component are within 4 steps of ‘Real number’. (This is for the network viewed as undirected; when viewed as directed, there is a direct path from ‘Real number’ to about about 83% of the space and 97.5% of these pages are within 5 steps of ‘Real number’.) The previous discussion strongly suggests that objective functions based on path-length do not seem likely to produce geodesics appropriate for knowledge space exploration.

It is important to remember that the metric space structure rides “on top of” the hyperlink structure. Once a candidate path between concepts is returned to a user, she still must navigate it using the hyperlinks. It is worth noting that the intentionally discursive (with respect to path length) traversal is very different from something like “targeted search” in a social network such as is taken up in [19]. If the “directions” on the network are well-chosen or defined vis-a-vis the individual nodes, then the mathematics of potential theory effectively forces a useful notion of distance between the concepts that are embodied in the nodes. Inherent in that are all the properties that come along with a (psuedo-) metric (e.g., symmetry and the triangle inequality). That said, this is but one of an infinity of metrics (and geometries) that could be imposed on the network and it is of interest to speculate on what kinds of objective functions would be useful for such ends, since, beyond the choice of weights and the sphere at infinity, with such an objective function one could decide between various candidate geometries for exploration.

## Methods

### The four-point probe on a network

Let 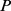 be the transition matrix of the ergodic Markov chain associated to our NWD. Although, here we work in the context of Markov chains [22], the four-point probe can be constructed in most places where potential theory makes good sense: see the Supporting Information for the construction on the Riemann Sphere). Let 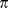 denote the equilibrium distribution for 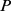 and let.
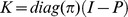
where 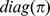 is the diagonal matrix with 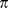 on the diagonal. The matrix 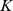 is the version of the Laplacian matrix that relates charge distribution 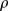 and electric potential 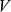 by
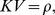
and as such we call it the *Kirchhoff Operator*. Note that 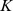 is not full rank and we require that the total charge is zero,
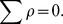
In the special case of reversible chains (networks with a symmetric weight matrix) this is electric network theory (see [5]), but we will use the same language/constructions for arbitrary chains.

Inspired by the idea of “plugging a battery into our network,” we place a positive unit charge at 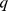, a negative unit charge at 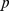, and we define 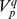 as the solution
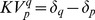
where 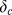 is point mass function (vector) concentrated at 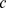. Thus, for every vertex 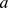, 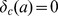 unless 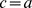 in which case it is equal to one. With this definition in hand, we can now define (and compute using linear algebra) our four-point probe on a network as
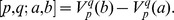



### The four-point probe metric

Recall that we define the *four-point probe metric* on our NWD by
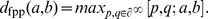



**Theorem 1** 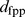 is a pseudometric.

**Proof:** This is a simple matter of checking off properties:

1. Non-negativity: 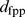 is non-negative since 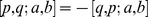, so the max is non-negative.
2. Symmetry: 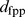 is symmetric since 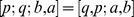, so 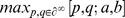 and 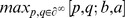 will agree.
3. Triangle inequality: To see 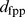 satisfies the triangle inequality first notice that 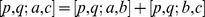 since
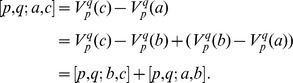

So using the 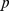 and 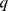 that maximize 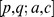, we have
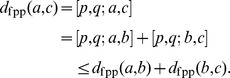



### Geodesic bundle consistency

As per the Results section we define *geodesic bundles* as 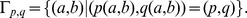



**Theorem 2** If 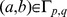 and 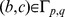, then 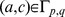.

**Proof:** We prove this by contradiction, and assume 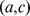 is not in 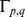. This implies 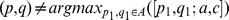
and so there exist 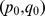 such that
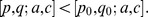



As in the proof of the triangle inequality in Theorem 1 and using the definition of the metric, we have
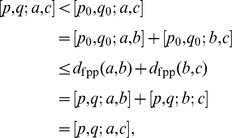
which is a contradiction.

### Data Sources

The MathWiki was extracted using a web crawler written in Python. This collected all the titles of the approximately 18,000 pages belonging to the Mathematics community in the English Wikipedia. For each page, we also recorded the set of links to other pages within the community. The code used to identify math articles is the List of Mathematics Articles page, which should be exhaustive for well-established pages following the Wikipedia article standards. This method of collecting the articles relies heavily on Wikipedia's user categorization scheme. The code is available here [34]. The final results and figures are from a run performed in April of 2013.

## Conclusion

In this paper we introduce the notion of the four-point probe geometry on a network and show that in the case in which we have a network with directions (NWD), defined as a network with a privileged set of vertices called directions, that we can define a new type of pseudometric on the network. We show that this pseudometric has properties much like that of a hyperbolic metric and its various analogies and similarities with the hyperbolic metric. In the case of a network with directions, we are able to define a notion of geodesic bundle that behaves much like the geodesics of hyperbolic geometry wherein we are able to formulate trajectories between points that go *from* a given direction *towards* another direction. In this way the directions act much like the points at infinity in these models. We show how the pages of Wikipedia devoted to Mathematics, the “MathWiki” describes a natural network with directions, in which the “List of” pages describe the set of directions. Through examples, we show how the geodesic currents give a more discursive, but natural means of navigating the *knowledge space* that is the MathWiki. Applications to other kinds of networks, including merchandise spaces are suggested as is the idea that such a metric could enable a network cartography well adapted to visual search.

## Supporting Information

Text S1 (PDF)
