# Supplementary material for: Orienteering in Knowledge Spaces: The Hyperbolic Geometry of Wikipedia Mathematics
Source: PLoS One. 2013 Jul 3;8(7):e67508. doi: 10.1371/journal.pone.0067508 (PMC3701066; doi:10.1371/journal.pone.0067508)
Supplement: Text S1 — (PDF) [file pone.0067508.s001.pdf]

Orienteering in Knowledge Spaces: The Hyperbolic Geometry of Wikipedia Mathematics - Supporting Information Text S1

G. Leibon[1,*], Daniel N. Rockmore[1,2,3]
1 Department of Mathematics, Dartmouth College, Hanover, NH, 03755 USA
2 Department of Computer Science, Dartmouth College, Hanover, NH, 03755 USA
3 The Santa Fe Institute, Santa Fe, NM, 87501 USA
∗ E-mail: gleibon@gmail.com

Supporting Information Text S1

The hyperbolic analogy

The four-point probe geometry for a network with directions shares much in common with models of hyperbolic geometry. Here we state the theorems we use to justify our hyperbolic analogies. As the results are germane to the spirit of the paper and the terminology we use, but not necessary for the results, we have placed them here in the Supporting Information. These results and their proofs involve only standard ideas in hyperbolic geometry (see e.g., [1]). In this section, we let H be the the interior of the closed unit disk D^2 and $\partial^\infty = \partial D^2$, considered with the usual hyperbolic (Poincaré) metric. Recall that this is isometric to the model of hyperbolic geometry given by the "Riemann sphere," the upper half plane of the complex plane with boundary given by the the real line and an ideal point at infinity, denoted ∞, which provides a compactification of the space. In either setting we make the same definition of $[p, q; a, b]$, that is, as the potential difference between a and b (in H) resulting from placing a charge of $+1$ at p and -1 at q (in ∂^∞). For notions of electric fields in these context see [2, 3].

The following theorems provide the analogies between our NWDs and these models .

Theorem S1. *Up to a global constant, the Poincaré metric on H is equal to*

$$max_{p,q\in\partial^\infty}[p, q; a, b].$$

Theorem S2. *Let $a, b \in H$ and*

$$(p(a, b), q(a, b)) = argmax_{p,q\in\partial^\infty}[p, q; a, b].$$

In the Poincaré model, the extended oriented geodesic connecting a to b tends towards $q(a, b)$, while the extended oriented geodesic connecting b to a tends towards $p(a, b)$.

Central to the proof of these theorems is the *cross-ratio*

$$\{p, q, a, b\} = \frac{(p - a)(q - b)}{(q - a)(p - b)}$$

of four points on the Riemann sphere

The cross ratio is related to the four-point probe $[p, q; a, b]$ as follows:

Lemma S1.

$$[p, q; a, b] = V_p^q(b) - V_p^q(a) = \frac{-1}{2\pi}\log|\{p, q, a, b\}|$$

where $\{p, q, a, b\}$ is the cross-ratio.

Proof: On the Riemann sphere we define the Kirchoff operator as

$$K = -\left(\frac{\partial^2}{\partial x^2} + \frac{\partial^2}{\partial y^2}\right).$$

It is well known that on the Riemann sphere that the Green's function, G_q, of a unit electric point charge where

$$KG_q = (\delta_q - \delta_\infty)$$

is given by

$$G_q = \frac{-1}{2\pi} \log |q - z|$$

(see [3]) . Via superposition we have

$$KV_p^q = (\delta_q - \delta_\infty) - (\delta_p - \delta_\infty).$$

hence the potential difference

$$V_p^q(z) = \frac{1}{2\pi} \log \left| \frac{q-z}{p-z} \right|.$$

and

$$V_p^q(b) - V_p^q(a) = \frac{1}{2\pi} \log |\{p, q, a, b\}|.$$

as needed.

■

The Poincaré (hyperbolic) metric on H is now easily related to $[p, q; a, b]$.

Lemma S2. *The Poincaré metric on H is equal to $2\pi[p, q; a, b]$ where p and q are the points where the geodesic through a and b intersect ∂^∞.*

Proof: The key fact is that the isometries in the Poincaré and upper half space models are precisely the conformal self-maps of H, and the cross-ratio is an invariant of the conformal self-maps. It is convenient to map the unit disk H to the upper half-plane model. Now the isometries are transitive on the unit tangent bundle, so up to a conformal transformation we may assume our points are $p = 0$, $q = \infty$, $a = i$, and $b = iy$. In this case, we can easily compute the distance since $\langle v, w \rangle_{hyp} = \frac{1}{y} \langle v, w \rangle_{euc}$ so that the distance from i to iy is $\log(y)$. Since the cross-ratio is a conformal invariant and $y = \{0, \infty, i, iy\}$ we now have the distance is $\log |\{p, q; a, b\}|$ as desired.

■

Proof (of Theorem S1): In the Poincaré disk model, the geodesic between a and b is a circular arc that intersects the ∂^∞ (the unit circle) at right angles. Furthermore, from Lemma S2 we have that the distance is $\log |\{p, q, a, b\}|$ where p and q are the points where this geodesic intersects ∂^∞. So all we need to show is that among all points $z, w \in \partial^\infty$ that p and q maximize the $\log |\{z, w, a, b\}|$. Using the key fact as stated in Lemma S2 and the fact the hyperbolic isometries are transitive on the unit tangent bundle, we may assume that $p = -1$, $q = 1$, $a = 0$, and $b = x$ with $0 < x < 1$, so that the geodesic is the "diameter" of D given by the embedded interval $[-1, 1]$. Any choices of z and w can be written as $z = e^{i\phi}$ and $w = e^{i\theta}$, and so we need to show that

$$\begin{aligned} \log |\{z, w, 0, x\}| &= \log \left| \tfrac{(e^{i\theta} - 0)(e^{i\phi} - x)}{(e^{i\theta} - x)(e^{i\phi} - 0)} \right| \\ &= \log \left| e^{i\phi} - x \right| - \log \left| (e^{i\theta} - x) \right| \end{aligned}$$

is maximized at $p = -1$ and $q = 1$. But since $|z - x|$ is the Euclidean distance from x to z and log monotonically increases, looking at the circle we see that is indeed true.

■

Proof (of Theorem S2): This now follows, effectively as a corollary of Theorem S1.

■

References

1. J. W. Cannon, W. J. Floyd, R. Kenyon, and W. R. Parry, Hyperbolic geometry, in *Flavors of Geometry*, pp. 59–115, Volume 31, MSRI Publications, 1997.

2. L. Ahlfors, *Complex Analysis*, McGraw-Hill, NY 1979.

3. P. Doyle and J. Steiner, Commuting time geometry of Markov chains, arXiv:1107.2612v1 [math.PR], 2011.
